# Gastroesophageal reflux disease at any cost: a dangerous paediatric attitude

**DOI:** 10.1111/j.1651-2227.2011.02315.x

**Published:** 2011-10

**Authors:** Andrea Taddio, Chiara Bersanini, Lucio Basile, Massimo Fontana, Alessandro Ventura

**Affiliations:** 1 Department of Pediatrics, Institute of Child Health IRCCS Burlo Garofolo, University of TriesteTrieste, Italy; 2 SCO Pediatria, Ospedale dei Bambini “V. Buzzi”Milan, Italy; 3 A.S.L. 105Pescara, Italy

Infantile spasm (IS) is a specific rare type of seizure seen in an epilepsy syndrome of infancy; it is also known as West syndrome and is characterized by developmental regression and a specific pattern on electroencephalography (EEG) testing called hypsarrhythmia (chaotic brain waves). Gastroesophageal Reflux Disease (GERD) is considered a common clinical problem in every day paediatrics clinical practice. However, GERD diagnosis seems to be overestimated, mainly because such symptoms as crying, regurgitation, feeding refusal, back arching, wheezing, coughing and hoarseness are still considered suggestive for GERD, although they have already been demonstrated to be inaccurate (1), with consequent risk for disease overdiagnosis and inappropriate unnecessary Proton Pump Inhibitors (PPI) prescription.

Herein, we report the cases of three patients with classical symptoms referable to IS who were initially misdiagnosed as having GERD.

## Case 1

A newborn presented a history characterized by short episodes of crying associated with slight neck flexions and stiffening of the arms. Events tended to occur after feeding. Diagnosis of GERD was made, and PPI therapy started. However, as introducing treatment, no improvement was noted, on the contrary clinical manifestations exacerbated in the following 2 months: The child become even more irritable, jerks presented more frequently, occurring also while sleeping, neck flexions became more prominent as well as arms attacks. Flexion movements of legs were noticed as well.

For this reason, the patient was admitted to our Gastroenterology Department.

Infant's rate of growth was normal. Physical examination was totally unremarkable except for signs of psychomotor development regression: The child stopped to smile in response to face and to reach for objects. Medical history and physical examination were consistent with the diagnosis of IS. Neurological assessment was requested, and an EEG showed a hypsarrhythmic pattern, pathognomonic of IS. Brain magnetic resonance imaging (MRI) did not show any pathological finding confirming the diagnosis of cryptogenic IS. Adenocorticotrope hormone (ACTH) therapy was started with clinical improvement.

## Case 2

A young baby was admitted for a 2-month history characterized by episodes of crying associated with flexion and extension movements of neck, arms and legs. Events occurred around sleeping and feedings. Clinical manifestations were initially classified as infant colics. However, after 3 weeks, the symptoms worsened (increasing in episodes frequency and duration, infant irritability) and were considered as suggestive for the diagnosis of GERD. PPI treatment was then started. Two months later, for persistence of symptoms, the child was admitted to our department. At admission, physical examination was negative, but the child presented extremely irritable and also in this case signs of psychomotor development regression were present: The infant could not show behaviour competences previously acquired like smiling on social contact, following objects with visual, sitting with supports.

Medical history and physical examination were consistent with IS diagnosis. EEG showed a hypsarrhythmia pattern confirming diagnosis. Neurological examination and brain MRI scans were completely unremarkable. Cryptogenic IS was diagnosed. Therapy with steroids and ACTH was started with marked improvement on clinical symptoms.

## Case 3

A 2-month-old boy who previously underwent surgical correction for intraventricular defect started to present frequent episodes of inconsolable crying, associated with neck extension, stiffening of the arms and trunk, sometimes followed by swallowing and vomiting.

Symptoms were attributed to GERD, and therapy with PPI started. However, no improvement was noticed. In the following weeks, episodes became even more frequents with exacerbation of symptoms. His mother video recorded an attack (Video S1), which showed the presence of flexion spasms, presence of intractable GERD was suggested and the child eventually underwent fundoplicatio.

No improvement was noticed: Persistence of symptoms and occurrence of spasms of the arms during acute episodes finally suggested diagnosis of West syndrome. IS was then confirmed by EEG while MRI was negative. Despite starting of appropriate therapy at 8 month of age, clinical signs of mental retardation were present.

Although IS is a rare disorder, diagnosis is usually not hard: The age of patients is peculiar and a careful patient history with a complete neurological evaluation are usually sufficient conditions to suspect the disease. Traditionally, differential diagnosis of IS does not contemplate GERD (2).

We have described the cases of three infants presenting with classical clinical findings attributable to IS who were initially diagnosed, and consequently treated, as having GERD. In all patients, irritability, spasms and crying were considered signs of gastrointestinal complaints rather than to psychomotor development regression.

To our knowledge, this is the first report of IS misdiagnosed as GERD.

We would like to underline that our patients presented classical signs of IS, and a more precise physical examination would have also revealed a psychomotor development regression. These findings were very consistent with IS that usually begins between age of 4 and 8 months and are characterized by brief symmetric contractions of neck, trunk and extremities. Spasms occur during sleep or arousal and have tendency to develop while patients are drowsy or immediately on awakening. These findings should always drive paediatricians to request for EEG, in which pattern most commonly associated with IS is hypsarrythmic.

We have tried to examine the reasons underlying such an exceptional misinterpretation.

Considering that both IS and GERD have not been changed their clinical presentation among past years, we believe that our report could be consider as the consequence of an extraordinary attention around GERD in the last decade.

In confirmation of this statement, Barron et al. have recently demonstrated that PPI use in paediatric population increased steadily from 1999 to 2004 in the United States (3).

We could speculate that a possible danger of such an attention towards GERD in children could be the erroneous attribution to GERD of symptoms clearly associated with other conditions like in patients we have described. Interestingly, it has already been shown that most of the symptoms classically related to GERD have revealed to be inconsistent with the clinical suspicion. In fact, only less of 10% of the symptoms conventionally associated with GER episodes (crying, regurgitation, feeding refusal, back arching, wheezing, coughing and hoarseness) are truly related to episodes of gastroesophageal reflux (GER) when detected with combined pH monitoring and impedance measurement (1). Similarly, Orenstein et al. (4) did not find any difference in efficacy between lansoprazole and placebo for symptoms attributed to GERD in infants.

We have already suggested that these symptoms, if not associated with other major complaint as growth failure or psychomotor development regression, should be simply considered part of the physiological behaviours of infants, rather than pathological events (5). Unfortunately, most paediatricians dealing with irritable infants still seem to be more prone to start with anti-reflux therapy rather than to educate parents on how to cope with infant crying as a part of anticipatory guidance. In our experience, this unjustified and ineffective approach may confuse the family, leading at the end to food refusal in the baby, with an impact on growth (6).

On the other hand, this particular attention to GERD in children may reduce attention towards other peculiar clinical conditions, like in cases we presented. We would like to underline that IS should be always considered in every infant presenting with symmetric spasms and psychomotor development regression.

In conclusion, we have described for the first time three cases of children affected by IS initially misdiagnosed with GERD. We believe that it could be the result of a peculiar overestimation of clinical signs attributable to GERD, beard from a flourishing specific literature in the past years, leading to overdiagnosis of GERD in healthy infants and to lack diagnosis of other specific clinical conditions like in cases we have presented.

In particular, IS long-term overall prognosis is poor, and mainly among those patients with long time from onset to treatment, cyptogenetic IS and age of onset <4 months, thereby prompt diagnosis is mandatory to start proper therapy.

We would suggest to consider GERD only in those patients with high risk rate for developing GERD like children with cerebral palsy, in those with surgery complications after oesophageal atresia and/or in any child with very evocative GERD symptoms, such as hematemesis and or chronic vomiting with growth failure.

## SUPPORTING INFORMATION

**Video S1** Epileptic attack in a patient affected by West syndrome.
